# Maternal pre- and perinatal depression and the risk of autism spectrum disorders in offspring: systematic review and meta-analysis

**DOI:** 10.1192/bjo.2025.67

**Published:** 2025-06-04

**Authors:** Biruk Shalmeno Tusa, Rosa Alati, Getinet Ayano, Kim Betts, Adisu Birhanu Weldesenbet, Berihun Dachew

**Affiliations:** School of Population Health, Curtin University, Perth, Western Australia, Australia; Department of Epidemiology and Biostatistics, College of Health and Medical Sciences, Haramaya University, Haramaya, Ethiopia; School of Public Health, The University of Queensland, Brisbane, Queensland, Australia; enAble Institute, Curtin University, Perth, Western Australia, Australia

**Keywords:** Antenatal depression, postnatal depression, pre-pregnancy depression, autism spectrum disorder (ASD)

## Abstract

**Background:**

Studies have reported conflicting findings on the association between maternal pre- and perinatal depression and autism spectrum disorder (ASD) in offspring.

**Aims:**

To examine and consolidate the existing evidence on the association between maternal pre- and perinatal depression and the risk of ASD in children and adolescents.

**Method:**

In this systematic review and meta-analysis, we searched PubMed, Medline, EMBASE, Scopus, CINAHL and PsycINFO from the database inception to 21 February 2024. A meta-analysis was performed using random-effect models, and summary effect estimates were presented as odds ratios with 95% confidence intervals. Heterogeneity was assessed with Cochran’s *Q* and the *I* ^2^-statistic test. Additionally, subgroup analysis was conducted to identify the source of potential heterogeneity within the included studies. A funnel plot and Egger’s regression test were employed to evaluate publication bias.

**Results:**

Twelve studies involving over 1.6 million mother–offspring pairs were included in the final analysis. A random-effects meta-analysis of these studies revealed a 52% increased risk (odds ratio 1.52, 95% CI 1.13–1.90) of ASD in the offspring of mothers experiencing pre-pregnancy depression, a 48% increased risk (odds ratio 1.48, 95% CI 1.32–1.64) in those experiencing antenatal depression and a 70% increased risk (odds ratio 1.70, 95% CI 1.41–1.99) in those with postnatal depression.

**Conclusions:**

This systematic review and meta-analysis found that offspring born to mothers with depression before, during and after birth have a higher risk of developing ASD. Our findings underscore the need for early screening and targeted intervention programmes for at-risk children.

Autism spectrum disorder (ASD) is characterised by enduring impairments in reciprocal social communication, social interaction and the presence of restricted, repetitive patterns of behaviour, interests or activities.^1,2^ It represents a lifelong neurodevelopmental condition, with a global prevalence of 0.82% among children aged between 6 and 12 years,^3^ exhibiting a higher occurrence in males than females.^4^ ASD stands as one of the most debilitating developmental disorders and imposes a significant economic burden.^5^

The causes of ASD remain mostly unclear; however, existing evidence suggests it has a complex origin, involving a significant genetic element along with environmental, physical and psychological factors.^6–11^ Among these many risk factors, maternal depression is particularly noteworthy because it is relatively common, affecting 21% of women during pregnancy^12^ and 14% postnatally.^13^ Maternal depression may serve as an indicator of chronic psychological stress, capable of triggering inflammation during pregnancy.^14,15^ This inflammatory response may have implications for the neurodevelopment of the unborn child.^16^ For instance, antenatal depression has been correlated with elevated fetal cortisol levels, which contribute to alterations in fetal hypothalamic-pituitary-adrenal axis activity and brain function.^17^ Studies further suggest that antenatal depression may induce epigenetic dysregulation in the offspring’s hypothalamic-pituitary-adrenal pathways and serotonin transmission.^18^ This dysregulation has the potential to result in heightened offspring hypothalamic-pituitary-adrenal reactivity and alterations in brain serotonin levels, ultimately influencing the neurodevelopment of the offspring and raising the risk of ASD.^19^

The availability of evidence concerning the risk of ASD in offspring with mothers experiencing depression is crucial for timely and suitable interventions for at-risk children. However, there is a notable lack of consistency in the findings of existing epidemiological studies. Although some studies have demonstrated links between maternal pre- and perinatal depression and an elevated risk of ASD in offspring,^20–28^ others found no increased risk.^29–31^ For instance, although a large prospective cohort study conducted in Taiwan by Chen et al^21^ identified more than a twofold increase in ASD risk among offspring of mothers with depression, a retrospective cohort study in the USA by Brennan et al^29^ found no association between maternal antenatal depression and ASD in offspring. Moreover, one systematic review and meta-analysis conducted 5 years ago reported the risk of developing ASD in offspring exposed to maternal depression, but its findings were based on three studies and were limited to antenatal exposure.^32^ These discrepancies, coupled with the absence of updated evidence on the subject, underscore the necessity for comprehensive evidence to address conflicting findings within the current literature and provide up-to-date pooled estimates. Therefore, this systematic review and meta-analysis aims to fill these gaps by thoroughly investigating the relationship between maternal pre- and perinatal depression and the risk of ASD in children and adolescents. The findings from this review have significant potential to inform preventive strategies, clinical guidelines and public health initiatives targeting maternal mental health support, optimal neurodevelopment promotion and the reduction of ASD risk in offspring.

## Method

### Study design

The results of this systematic review and meta-analysis were reported in accordance with the guidelines outlined by the Preferred Reporting Items for Systematic Review and Meta-Analysis Statement (PRISMA).^33^ Additionally, the protocol for conducting this systematic review and meta-analysis was pre-registered in the International Prospective Register of Systematic Reviews and Meta-analysis (PROSPERO) under the registration number CRD42023420211 (see Supplementary Table 1).

### Data sources and searches

We conducted a comprehensive search for relevant articles across six databases, including PubMed, Medline, EMBASE, Scopus, CINAHL and PsycINFO, covering the period from the inception of each database to 21 February 2024. The search employed appropriate subject headings and keywords related to ASD and maternal depression. These included (‘neurodevelopmental disorder’ OR ‘Autis*’ OR ‘autism spectrum disorder*’ OR ‘autistic disorder*’ OR ‘ASD’ or ‘Asperger syndrome’ OR ‘pervasive developmental disorder*’ OR ‘Rett’s syndrome’ OR ‘childhood disintegrative disorder*’) AND (‘Maternal depression’ OR ‘prenatal depression’ OR ‘postnatal depression’ OR ‘postpartum depression’ OR ‘antenatal depression’). In addition to the database searches, we reviewed the reference lists of included studies to identify any additional relevant studies. The search strategy was formulated with the assistance of a medical librarian, and a comprehensive list of all search terms can be accessed in the supplementary information (see Supplementary Table 2).

### Eligibility criteria

We included all observational studies (case–control and cohort) that investigated maternal depression as the exposure variable, irrespective of the timing (before and during pregnancy, or after childbirth). These studies were required to assess ASD as an outcome in children and adolescents. Eligible studies reported relevant statistical measures, such as odds ratios, hazard ratios and relative risks, along with their associated 95% confidence intervals, or provided data allowing us to calculate these effect estimates. There were no restrictions on the publication year, and we considered studies published in English for inclusion. Conversely, animal studies, case reports, case series, correspondence, abstracts and reviews were excluded from consideration.

### Definition outcome and exposure variables

The study considered ASD as the dependent variable, which is determined according to established diagnostic criteria such as the DSM^34^ and the ICD.^35^ Maternal depression, whether occurring before, during or after the pregnancy period, was treated as the independent variable and assessed by well-recognised tools for diagnosing or screening for depression, such as the ICD, DSM, Edinburgh Postnatal Depression Scale (EPDS) and the Center for Epidemiological Studies Depression Scale (CES-D).

### Data extraction

We began by identifying relevant studies and importing them into our citation manager (Microsoft EndNote version 20 for Windows, Clarivate, Philadelphia, PA, USA; see https://endnote.com/downloads/), ensuring the removal of any duplicate entries. Subsequently, we used Rayyan software (Rayyan Systems, Cambridge, MA, USA; see https://www.rayyan.ai/) to conduct title and abstract screening. Two reviewers (B.T. and A.B.W.) independently evaluated titles, abstracts and full texts to identify articles that met our eligibility criteria. Any disagreements between the two reviewers were resolved through discussion.

These same two reviewers then independently extracted data from the included articles on 28–29 February 2024. The extraction process entailed recording details such as the first author’s name, year of publication, country of study, study design, sample size, types of maternal depression, instruments used for measuring exposures and outcomes, period of outcomes measured, confounders adjusted for, and measures of effect (including odds or hazard ratios, or relative risks) along with their respective 95% confidence intervals. In instances where studies provided multiple estimates, preference was given to those that underwent the most comprehensive adjustments. When studies presented various types of outcomes or measured a single outcome at different exposure times, each was treated as a distinct set of data.

### Study quality appraisal

We utilised the Newcastle–Ottawa Scales (NOSs) to evaluate the methodological quality and risk of bias in studies.^36^ These tools incorporated specific criteria, including the selection of study groups (4 points), comparison of groups (2 points) and ascertainment of the desired outcome (3 points). The scoring system for these tools ranged from 0 to 9, allowing the categorisation of studies into low-quality (1–4), medium-quality (5–7) and high-quality (8–9) groups. The assessment of study quality was independently conducted by B.T. and A.B.W., with any discrepancies resolved through discussion between them.

### Data synthesis and statistical analysis

We conducted a meta-analysis using random-effect models to synthesis the available evidence. Our summary effect estimates were presented with the odds ratio accompanied by 95% confidence intervals. To assess statistical heterogeneity across the included studies, Cochran’s *Q* and the *I* ^2^-statistic test were utilised.^34,37^ Furthermore, subgroup analyses were conducted to examine differences in outcomes based on specific criteria, including time and measure of exposure, study design, year of publication, quality of study and some adjusted factors. To investigate the possibility of publication bias, we examined funnel plots and conducted Egger’s regression test.^38^ All analyses were carried out with R software version 4.3.1 for Windows (The R Project, The R Foundation, Vienna, Austria; see https://cran.r-project.org/bin/windows/base).

## Results

### Search results

**Fig. 1:**
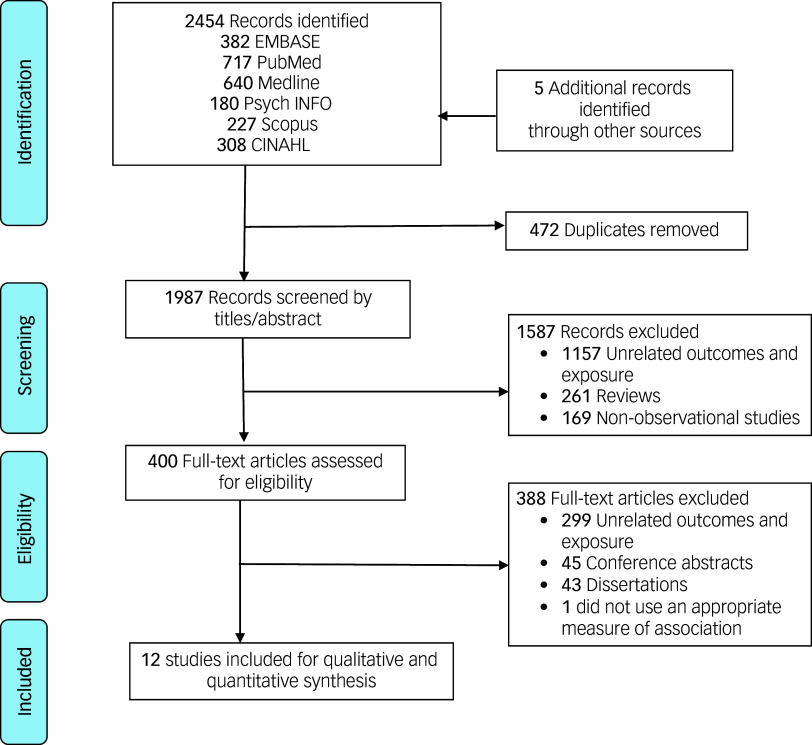
The PRISMA flow diagram. PRISMA, Preferred Reporting Items for Systematic Review and Meta-113 Analysis.

The search strategy and manual search identified 2459 records in the database search. After eliminating duplicates, a total of1987 records were screened based on their titles and abstracts for eligibility. Among these, 400 publications were found to be suitable for a full-text review. Finally, 12 studies were included in this systematic review and meta-analysis (Fig. 1).^20–31^

### Characteristics of included studies

Table 1 provides key characteristics of the studies included in this systematic review and meta-analysis. A total of 12 articles, comprising 1 655 966 mother–offspring pairs, were included.^20–31^ These studies were conducted from 2011 to 2023 across seven different countries. Five of the studies were conducted in the USA,^20,22,29–31^ two in Turkey^24,28^ and another two in Denmark.^23,26^ Among the studies included in our analysis, eight were case–control studies^22–25,27,28,30,31^ and the remaining four were cohort studies.^20,21,26,29^ All studies utilised diagnostic tools such as the ICD and DSM to assess ASD. Additionally, six studies employed these tools to evaluate maternal depression.^20–22,27,30,31^ Out of the 12 studies analysed, nine reported antenatal depression,^20,21,24–30^ three focused on postnatal depression^21,24,28^ and five investigated pre-pregnancy depression as the exposure variable.^21–23,30,31^

**Table 1:** Characteristics of included studies

Author, year	County of study	Study design	Sample size	Time of exposure	Measure of exposure	Offspring age at measurement	Measures of outcomes	Measures of effect
Avalos et al 2023^20^	USA	Cohort study	3994 (635 exposure and 3359 non-exposed)	Antenatal depression	ICD-9	12 years	SRS	Odds ratio 1.64, 95% CI 1.09–2.46
Brennan et al 2023^29^	USA	Cohort study	2377	Antenatal depression	Not reported	1.5–12 years	ADOS	Odds ratio 1.30, 95% CI 0.80–2.20
Castro et al 2016^30^	USA	Matched case–control	4650 (1455 cases and 3405 controls)	Pre-pregnancy depression	ICD-9	2–19 years	ICD-9	Odds ratio 1.21, 95% CI 0.87–1.66
				Antenatal depression		2–19 years		Odds ratio 1.32, 95% CI 0.93–1.84
Chen et al 2020^21^	Taiwan	Cohort study	708 515 (53 489 exposed and 655 026 non-exposed)	Pre-pregnancy depression	ICD-9	0–11 years	ICD-9	Hazard ratio 2.01, 95% CI 1.70–2.37
				Antenatal depression		0–11 years		Hazard ratio 1.58, 95% CI 1.11–2.25
				Postnatal depression		0–11 years		Hazard ratio 1.64, 95% CI 1.52–1.76
Clements et al 2015^22^	USA	Matched case-control	5399 (1377 cases and 4022 controls)	Pre-pregnancy depression	ICD-9	2–19 years	ICD-9	Odds ratio 1.74, 95% CI 1.35–2.23
Croen et al 2011^31^	USA	Unmatched case–control	1805 (298 cases and 1507 controls)	Pre-pregnancy depression	ICD-9	3.6–3.8 years	ICD-9	Odds ratio 0.84, 95% CI 0.21–1.48
				Antenatal depression				Odds ratio 1.04, 95% CI 0.34–1.73
Gidaya et al 2014^23^	Denmark	Matched case–control	57 365 (5215 cases and 52 150 controls)	Pre-pregnancy depression	Not reported	Not reported	ICD-9	Odds ratio 1.59, 95% CI 1.19–2.10
Güneş et al 2023^24^	Turkey	Matched case–control	452 (211 cases and 241 controls)	Antenatal depression	Not reported	2–6 years	DSM-V	Odds ratio 3.26, 95% CI 1.02–10.39
				Postnatal depression				Odds ratio 2.04, 95% CI 1.35–3.08
Hagberg et al 2017^25^	UK	Matched case–control	196 648 (2154 cases and 194 494 controls)	Antenatal depression	Not reported	0–8 years	Read diagnostic code	Relative risk 1.49, 95% CI 1.27–1.75
Hviid et al 2013^26^	Denmark	Cohort study	626 875 (3482 exposure and 623 393 non-exposed)	Antenatal depression	No tools are used	4–7.5 years	ICD-10	Relative risk 1.82, 95% CI 1.32–2.52
Rai et al 2013^27^	Sweden	Matched case–control	47 706 (4429 cases and 43 277 controls)	Antenatal depression	ICD-10	0–17 years	ICD-10	Odds ratio 1.49, 95% CI 1.08–2.08
Say et al 2016^28^	Turkey	Matched case–control	180 (100 cases and 80 controls)	Antenatal depression	Self-report	3–18 years	DSM-IV	Odds ratio 2.89, 95% CI 1.43–5.84
				Postnatal depression		3–18 years		Odds ratio 2.89, 95% CI 1.45–5.76

SRS, Social Responsiveness Scale; ADOS, Autism Diagnostic Observation Schedule; ICD, International Classification of Diseases; DSM, Diagnostic and Statistical Manual of Mental Disorders.

### Adjustment of confounder factors

Of the 12 studies, nine accounted for at least one confounding factor in their effect estimates.^20–22,25–27,29–31^ Eight studies considered the impact of socioeconomic factors^20–22,26,27,29–31^ and six adjusted for maternal substance use.^20,25–27,29,30^ Additionally, five studies accounted for antidepressant use, and four controlled for other psychiatric disorders.^25–27,30^ Notably, only two studies included adjustments for paternal depressive disorders (see Supplementary Table 3).^21,27^

### Quality assessment

All included studies were rated medium to high based on the NOS quality assessment, with a median score of 8 (interquartile range: 2) points. Eight studies achieved scores of eight or nine points, indicating high quality,^20–22,26–31^ whereas the remaining four received scores of 7 points, falling within the category of medium-quality studies (see Supplementary Table 4).^23–25^

### Maternal depression and risk of ASD

**Fig. 2:**
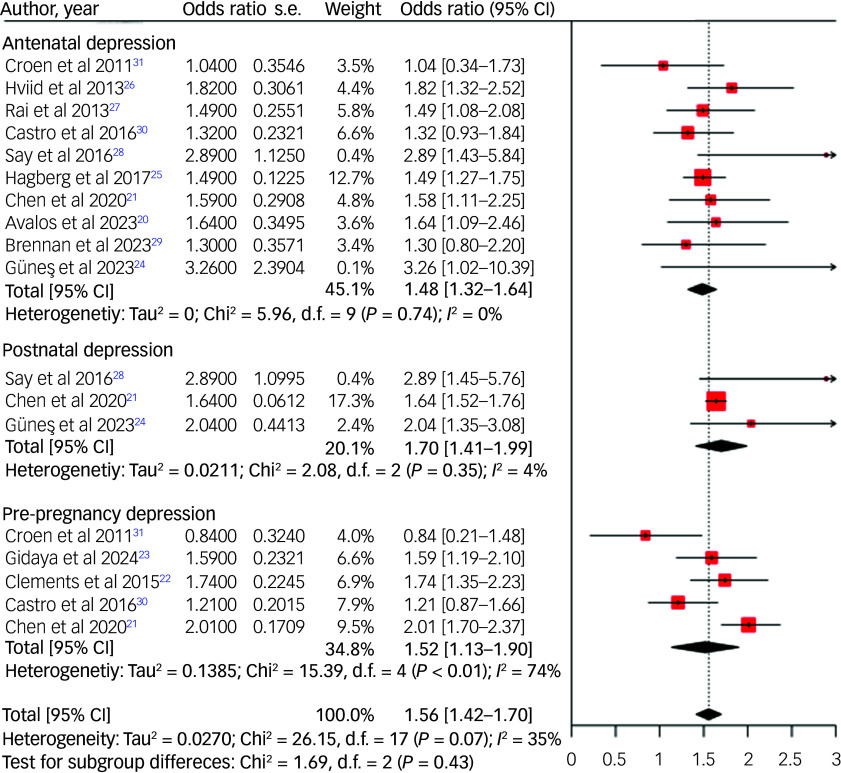
Forest plot for risk of autism spectrum disorder among offspring of mothers with depression.

Among the 12 studies examining the link between maternal pre- and perinatal depression and the subsequent risk of ASD, nine recorded significant relationships, whereas the remaining three found no evidence of associations. A random-effects meta-analysis of these studies revealed a 52% increased risk (odds ratio 1.52, 95% CI 1.13–1.90) of ASD in the offspring of mothers experiencing pre-pregnancy depression, a 48% increased risk (odds ratio 1.48, 95% CI 1.32–1.64) in those experiencing antenatal depression, and a 70% increased risk (odds ratio 1.70, 95% CI 1.41–1.99) in those experiencing postnatal depression (Fig. 2).

### Subgroup analysis

A stratified analysis based on study design indicated a higher risk of offspring ASD in cohort studies (odds ratio 1.67, 95% CI 1.56–1.78) compared with case–control studies (odds ratio 1.45, 95% CI 1.31–1.59). Additionally, our subgroup analysis identified a greater risk of offspring ASD in studies published between 2017 and 2023 (odds ratio 1.66, 95% CI 1.49–1.83) compared with those from 2011–2016 (odds ratio 1.44, 95% CI 1.23–1.64). Positive associations between maternal depression and ASD in offspring were noted in studies employing both diagnostic instruments (odds ratio 1.60, 95% CI 1.39–1.76) and screening instruments (odds ratio 1.57, 95% CI 1.39–1.76). Similarly, a positive association was evident in both high-quality (odds ratio 1.59, 95% CI 1.50–1.69) and medium-quality studies (odds ratio 1.57, 95% CI 1.36–1.77).

The risk of ASD was slightly lower in studies that accounted for at least one confounding factor (odds ratio 1.52, 95% CI 1.36–1.68) compared with those that did not (odds ratio 1.77, 95% CI 1.39–2.16). Specifically, the risk of offspring ASD exhibited a slight decrease in studies that accounted for the effects of socioeconomic factors (odds ratio 1.61, 95% CI 1.52–1.71 *v.* odds ratio 1.53, 95% CI 1.34–1.71), other maternal psychiatric disorders (odds ratio 1.62, 95% CI 1.52–1.71 *v.* odds ratio 1.49, 95% CI 1.31–1.68), maternal substance use (odds ratio 1.49, 95% CI 1.32–1.66 *v.* odds ratio 1.58, 95% CI 1.35–1.81) and maternal antidepressant use (odds ratio 1.40, 95% CI 1.22–1.58 *v.* odds ratio 1.65, 95% CI 1.55–1.7) compared with those that did not adjust for these factors. Only two studies accounted for paternal depression in their analysis. These studies reported a higher risk of ASD (odds ratio 1.67, 95% CI 1.56–1.78) compared with those that did not account for paternal depression (odds ratio 1.47, 95% CI 1.33–1.61). It is important to note that both of these studies exhibited high ratings in the NOS quality assessment, utilised substantial sample sizes and employed validated diagnostic tools (ICD-9/10) to assess the outcome (ASD) and exposure (maternal depression). These methodological strengths likely contributed to the higher risk estimates observed in this subset of studies (Table 2).

**Table 2:** Subgroup analysis on risk of autism spectrum disorder in offspring of mothers with depression

Subgroups	Number of studies	Pooled odds ratio (95% CI)	Heterogeneity across the studies (*I* ^2^)	Heterogeneity between the groups (*P*-value)
Exposure measures				
Screening	5	1.57 (1.39–1.76)	0.0%	0.64
Diagnostic	7	1.60 (1.50, 1.69)	54.9%	
Study design				
Cohort	4	1.67 (1.56–1.78)	9.9%	0.03
Case–control	8	1.45 (1.31–1.59)	24.1%	
Year of publication				
2011–2016	7	1.44 (1.23–1.64)	33.1%	0.10
2017–2023	5	1.66 (1.49–1.83)	16.8%	
Quality of study				
Medium	4	1.57 (1.36–1.77)	0.0%	0.72
High	8	1.59 (1.50–1.69)	47.9%	
Adjusted for any confounding factors				
Yes	9	1.52 (1.36–1.68)	49.0%	0.20
No	3	1.77 (1.39–2.16)	0.0%	
Adjusted for socioeconomic factors				
Yes	8	1.61 (1.52–1.71)	49.2%	0.95
No	4	1.53 (1.34–1.71)	0.0%	
Adjusted for other maternal psychiatric disorders				
Yes	4	1.62 (1.52–1.71)	43.6%	0.57
No	8	1.49 (1.31–1.68)	0.0%	
Adjusted for maternal substance use				
Yes	6	1.49 (1.32–1.66)	0.0%	0.52
No	6	1.58 (1.35–1.81)	50.5%	
Adjusted for antidepressant use				
Yes	5	1.40 (1.22–1.58)	19.3%	0.04
No	7	1.65 (1.55–1.75)	24.7%	
Adjusted for paternal depressive disorders				
Yes	2	1.67 (1.56–1.78)	37.4%	0.07
No	10	1.47 (1.33–1.61)	19.7%	

### Publication bias

**Fig. 3:**
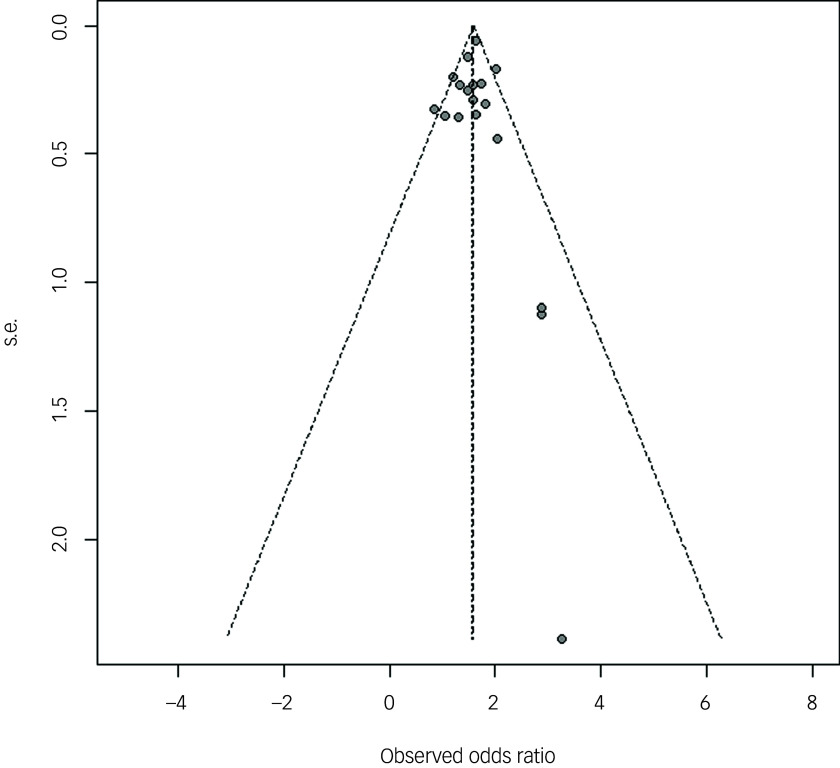
Publication bias of studies.

The symmetrical funnel plots and Egger’s test indicate no evidence of substantial publication bias among the included studies (*P* = 0.92) (Fig. 3).

## Discussion

In our systematic review and meta-analysis, we investigated the potential association between maternal pre- and perinatal depression and the risk of ASD in offspring, based on 12 studies involving over 1.6 million mother–offspring pairs. We found that offspring exposed to maternal pre-conception, antenatal and postnatal depressions exhibited a 48–70% increased risk of ASD. The observed associations were independent of methodological factors such as ascertainment of exposure and outcome, year of publication, study quality and adjustment for important potential confounders, including socioeconomic status, maternal substance use, comorbid maternal psychiatric disorders, paternal depression and maternal antidepressant use.

Although the exact mechanism elucidating the observed associations between pre- and perinatal depression and the elevated risk of ASD in offspring remains unclear, several interpretations have been proposed. First, it is suggested that there may exist a shared genetic predisposition between depression and ASD, where certain genetic variations or mutations could contribute to both conditions, thus increasing the probability of their co-occurrence within families.^11^ Second, epigenetic factors, such as alterations in DNA methylation patterns, might play a role in mediating the relationship between maternal depression and the risk of ASD in offspring, implying environmental influences on gene expression.^18^ Maternal depression during pregnancy could potentially affect DNA methylation of the fetus through changes in maternal stress hormones, inflammatory cytokines or neurotransmitter levels.^14,17,19^ These alterations could subsequently affect the developing fetal brain, thereby elevating the risk of ASD in the offspring.^15–17^

Maternal behaviour and caregiving practices may explain the observed associations between maternal postpartum depression and ASD risk. Maternal depression can affect the quality of maternal–child interactions,^39^ potentially worsening existing ASD symptoms in children.^40^ For instance, women who experience depression often face difficulties in bonding with their children or find it more challenging to respond to the added needs of children at risk of ASD.^39^ These challenges can extend to breastfeeding, as the emotional connection and interaction between mother and baby play a crucial role in successful breastfeeding.^41^ Consequently, suboptimal breastfeeding practices have been associated with the worsening of existing ASD symptoms in children.^42^ In addition, postpartum depression is often associated with chronic stress and dysregulated hypothalamic-pituitary-adrenal axis function, leading to elevated cortisol levels.^43,44^ Exposure to high levels of maternal cortisol during early infancy can affect the developing child’s stress response systems and neural circuits involved in emotional regulation and social behaviour, potentially increasing the risk of ASD.^45^ Postpartum depression is associated with immune dysregulation and increased inflammation.^46^ Maternal inflammatory markers and cytokines can also transmit through breast milk, exposing the infant to an inflammatory environment that may affect brain development and increase the risk of neurodevelopmental disorders, including ASD.^47,48^ In light of the above mechanisms, although the 95% confidence intervals for all three types of maternal depression overlap, suggesting that these differences are not statistically significant, the effect estimate is slightly higher for postnatal depression compared with pre-pregnancy and antenatal depression. Although antenatal depression is critical because of its potential effects on early brain development *in utero*, postnatal depression may exert additional influence through altered caregiving behaviours, disrupted maternal–infant bonding and increased environmental stressors during a crucial period of rapid brain growth and neural plasticity. Furthermore, postnatal depression may represent a continuation or exacerbation of earlier depressive states, compounding the overall risk to the child. Additionally, the number of studies included in the analyses could contribute to variability in the effect estimates. Although five studies were included for pre-pregnancy depression and ten for antenatal depression, only three studies were available for postnatal depression.

Another plausible explanation for the association between maternal depression and the risk of ASD is the potential confounding effect of maternal antidepressant use during pregnancy. Antidepressants, particularly selective serotonin reuptake inhibitors (SSRIs) and serotonin/noradrenaline reuptake inhibitors (SNRIs) can traverse the placental barrier.^49^ Research indicates that exposure to serotonergic agents *in utero* can induce enduring alterations in brain circuitry, diminish serotonergic reactivity and manifest behavioural traits akin to autism in animal models.^50^ Moreover, pregnant women who take SSRIs /SNRIs are more likely to have frequent medical consultations during and after pregnancy compared wuth those who do not. Consequently, their children may also have more medical encounters, potentially leading to a higher likelihood of ASD detection compared with children of non-exposed mothers. Our study’s estimates were notably influenced by adjustment for this factor, suggesting its significance in interpreting the association. A systematic review and meta-analysis comprising more than 100 000 mother–offspring pairs reported a positive association between antidepressant exposure and the risk of ASD.^51^ In our meta-analysis, we observed a notable decrease in the risk of ASD in studies that accounted for maternal antidepressant use, indicating a potential influence of these exposures on the observed association. It is also plausible that if women effectively manage their depression with antidepressants, they may be better equipped to handle their children’s condition, potentially leading to a reduced risk of ASD. However, this adjustment did not completely attenuate the association, suggesting that maternal antidepressant may have contributed only partly to the increased risk of ASD in offspring.

Socioeconomic factors, such as maternal age, maternal education status and family income, alongside substance use behaviours such as alcohol and cigarette consumption, are also significant confounders in the relationship between maternal depression and the risk of offspring ASD.^52–55^ Research has shown a link between maternal depression and lower socioeconomic status,^52^ with low socioeconomic status itself being associated with an increased risk of ASD in offspring.^53,54^ This relationship may be explained by the exposure of mothers to social stressors associated with low socioeconomic status, particularly during pregnancy or childbirth, which could affect ASD risk through epigenetic mechanisms.^15,17^ Furthermore, factors associated with lower socioeconomic status, such as lead poisoning, exposure to other neurotoxins and alcohol consumption during pregnancy, could also contribute to this relationship.^48^ Existing epidemiology studies suggested that substance use is often associated with maternal depression,^56^ and this substance use can have harmful effects on fetal brain development and increase the risk of ASD.^55^ Consistent with this, our meta-analysis revealed a slight decrease in ASD risk in studies that accounted for socioeconomic factors and substance use, suggesting a potential influence of these variables on the observed association. However, even after adjusting for these factors, the link between maternal depression and the risk of offspring developing ASD persisted.

There are also likely additional factors that could significantly influence the association between maternal depression and the risk of offspring ASD. However, these factors have not been adequately controlled for in most of the included studies. For example, of the 12 studies in our meta-analysis, only two adjusted for paternal depression.^21,27^ However, it is important to note that our subgroup analyses revealed positive associations in both studies that did and did not account for paternal depression, although the effect estimate is slightly higher for the former one. The difference in study design, sample size, study quality and ascertainment of exposure and outcome may have contributed to this disparity.

### Strengths and limitations of the study

This systematic review and meta-analysis includes several noteworthy strengths. It investigates the relationship between maternal pre- and perinatal depression, and the likelihood of ASD in offspring, offering a comprehensive overview of existing research. The exploration of sources of heterogeneity through subgroup analyses enhances the study’s credibility by identifying potential variations. Moreover, the high scores on the NOS for most included studies reflect a high methodological quality among the reviewed studies.

Despite its strengths, the study has notable limitations. Variations in how maternal depression were assessed may introduce errors in risk estimation, affecting validity. Although no evidence of publication bias was found, potential bias resulting from unpublished research remains. Additionally, several potential confounders, such as parental neurodevelopmental disorders, maternal physical comorbidities, prenatal health conditions and obstetric complications, were not accounted for in the majority of studies. As a result, the observed association between maternal depression and the increased risk of ASD in offspring must be interpreted with caution because of the insufficient or absent adjustment for these important factors in most included studies. Future research should address these and other unmeasured confounders to provide a more thorough and accurate understanding of the relationships. Moreover, given that the association between postnatal depression and the risk of ASD observed in this meta-analysis is based on only three studies, further research is needed to strengthen the evidence. Finally, the generalisability of findings may be limited as many studies were conducted in developed countries, limiting applicability to developing countries with different healthcare systems, cultural factors and socioeconomic conditions.

In conclusion, our systematic review and meta-analysis have revealed an elevated risk of ASD in the offspring of mothers who experience pre-and perinatal depression. Therefore, early screening and targeted intervention programmes for these at-risk children and adolescents are essential.

## Supporting information

Tusa et al. supplementary material Tusa et al. supplementary material

## Data Availability

The data that support the findings of this study are available from the corresponding author, B.T., upon reasonable request.

## Supplementary material

The supplementary material can be found online at https://doi.org/10.1192/bjo.2025.67
