# Supplementary material for: Maternal pre- and perinatal depression and the risk of autism spectrum disorders in offspring: systematic review and meta-analysis
Source: BJPsych Open. 2025 Jun 4;11(4):e117. doi: 10.1192/bjo.2025.67 (PMC12188230; doi:10.1192/bjo.2025.67)
Supplement: Tusa et al. supplementary material [file S2056472425000675sup001.docx]

**Contents**

[**eTable 1**. PRISMA-Statement to report the findings of the study. 3](#_Toc162478396)

[**eTable 2**. Search Terms and Strategy 5](#_Toc162478397)

[**eTable 3**. Confounder Variables Accounted by Studies Included in the Systematic Review and Meta-Analysis 6](#_Toc162478398)

[**eTable 4.** Quality Assessment of Studies Included in the Final Analysis According to the Newcastle-Ottawa Scale (NOS) 7](#_Toc162478399)

[**References** 9](#_Toc162478400)

# **eTable 1**. PRISMA-Statement to report the findings of the study.

| **Section/topic** | **#** | **Checklist item** | **Reported on page #** |
| --- | --- | --- | --- |
| **TITLE** | | |  |
| Title | 1 | Identify the report as a systematic review, meta-analysis, or both. | 1 |
| **ABSTRACT** | | |  |
| Structured summary | 2 | Provide a structured summary including, as applicable: background; objectives; data sources; study eligibility criteria, participants, and interventions; study appraisal and synthesis methods; results; limitations; conclusions and implications of key findings; systematic review registration number. | 2 |
| **INTRODUCTION** | | |  |
| Rationale | 3 | Describe the rationale for the review in the context of what is already known. | 4 |
| Objectives | 4 | Provide an explicit statement of questions being addressed with reference to participants, interventions, comparisons, outcomes, and study design (PICOS). | 4 |
| **METHODS** | | |  |
| Protocol and registration | 5 | Indicate if a review protocol exists, if and where it can be accessed (e.g., Web address), and, if available, provide registration information including registration number Web address: ***CRD42023420211*** | 4 |
| Eligibility criteria | 6 | Specify study characteristics (e.g., PICOS, length of follow-up) and report characteristics (e.g., years considered, language, publication status) used as criteria for eligibility, giving rationale. | 5 |
| Information sources | 7 | Describe all information sources (e.g., databases with dates of coverage, contact with study authors to identify additional studies) in the search and date last searched. | 5 |
| Search | 8 | Present full electronic search strategy for at least one database, including any limits used, such that it could be repeated. | 5 & eTable 2 in Supplement |
| Study selection | 9 | State the process for selecting studies (i.e., screening, eligibility, included in systematic review, and, if applicable, included in the meta-analysis). | 6 |
| Data collection process | 10 | Describe method of data extraction from reports (e.g., piloted forms, independently, in duplicate) and any processes for obtaining and confirming data from investigators. | 6 |
| Data items | 11 | List and define all variables for which data were sought (e.g., PICOS, funding sources) and any assumptions and simplifications made. | 6 |
| Risk of bias in individual studies | 12 | Describe methods used for assessing risk of bias of individual studies (including specification of whether this was done at the study or outcome level), and how this information is to be used in any data synthesis. | 7 |
| Summary measures | 13 | State the principal summary measures (e.g., risk ratio, difference in means). | 7 |
| Synthesis of results | 14 | Describe the methods of handling data and combining results of studies, if done, including measures of consistency (e.g., I^2^) for each meta-analysis. | 7 |
| Risk of bias across studies | 15 | Specify any assessment of risk of bias that may affect the cumulative evidence (e.g., publication bias, selective reporting within studies). | 7 |
| Additional analyses | 16 | Describe methods of additional analyses (e.g., sensitivity or subgroup analyses, meta-regression), if done, indicating which were pre-specified. | 7 |
| **RESULTS** | | |  |
| Study selection | 17 | Give numbers of studies screened, assessed for eligibility, and included in the review, with reasons for exclusions at each stage, ideally with a flow diagram. | 7 & Figure 1 |
| Study characteristics | 18 | For each study, present characteristics for which data were extracted (e.g., study size, PICOS, follow-up period) and provide the citations. | 8 & Table 1 |
| Risk of bias within studies | 19 | Present data on risk of bias of each study and, if available, any outcome level assessment (see item 12). | 8 & eTable 3 in Supplement |
| Results of individual studies | 20 | For all outcomes considered (benefits or harms), present, for each study: (a) simple summary data for each intervention group (b) effect estimates and confidence intervals, ideally with a forest plot. | 9 & Figure 2 |
| Synthesis of results | 21 | Present results of each meta-analysis done, including confidence intervals and measures of consistency. | 9 & Figure 2 |
| Risk of bias across studies | 22 | Present results of any assessment of risk of bias across studies (see Item 15). | 10 & Figure 3 |
| Additional analysis | 23 | Give results of additional analyses, if done (e.g., sensitivity or subgroup analyses, meta-regression [see Item 16]). | 9 & Table 2 |
| **DISCUSSION** | | |  |
| Summary of evidence | 24 | Summarize the main findings including the strength of evidence for each main outcome; consider their relevance to key groups (e.g., healthcare providers, users, and policy makers). | 10-14 |
| Limitations | 25 | Discuss limitations at study and outcome level (e.g., risk of bias), and at review-level (e.g., incomplete retrieval of identified research, reporting bias). | 14 |
| Conclusions | 26 | Provide a general interpretation of the results in the context of other evidence, and implications for future research. | 14 |
| **FUNDING** | | |  |
| Funding | 27 | Describe sources of funding for the systematic review and other support (e.g., supply of data); role of funders for the systematic review. | 15 |

# **eTable 2**. Search Terms and Strategy

| **Terms** | **CONCEPT 1** | **CONCEPT 2** |
| --- | --- | --- |
|  | **Maternal depression** | **Chid mental disorder** |
| **Keywords (Ovid)**  Medline, EMBASE & PsychINFO | ("Maternal depression" or "prenatal depression" or "postnatal depression" or "postpartum depression" or "antenatal depression").tw,ab. | ("mental disorder" adj5 (child* or offspring or adolescent or juvenile or young or teenager or youth or toddler)).tw,ab.  OR  ("neurodevelopmental disorder" or Autis* or "autism spectrum disorder*" or "autistic disorder*" or "ASD" or "Asperger syndrome" or "pervasive developmental disorder*" or "Rett's syndrome" or "childhood disintegrative disorder*") .tw,ab. |
| **Keywords (EBSCO)**  CINAHL | TI ("Maternal depression" or "prenatal depression" or "postnatal depression" or "postpartum depression" or "antenatal depression")  OR  AB ("Maternal depression" or "prenatal depression" or "postnatal depression" or "postpartum depression" or "antenatal depression") | TI ("mental disorder" N5 (child* or offspring or adolescent or juvenile or young or teenager or youth or toddler))  OR  AB ("mental disorder" N5 (child* or offspring or adolescent or juvenile or young or teenager or youth or toddler))  OR  TI ("neurodevelopmental disorder" or Autis* or "autism spectrum disorder*" or "autistic disorder*" or "ASD" or "Asperger syndrome" or "pervasive developmental disorder*" or "Rett's syndrome" or "childhood disintegrative disorder*")  OR  AB ("neurodevelopmental disorder" or Autis* or "autism spectrum disorder*" or "autistic disorder*" or "ASD" or "Asperger syndrome" or "pervasive developmental disorder*" or "Rett's syndrome" or "childhood disintegrative disorder*") |
| **Subject Headings** | | |
| **PubMed [Mesh]** | "Depression, Postpartum"[Mesh] | "Neurodevelopmental Disorder*"[Mesh] |
| **Medline (Ovid)** | exp Depression, Postpartum/ | exp Neurodevelopmental Disorders/ |
| **Embase (Ovid)** | exp postnatal depression/ | exp child psychiatry/ |
| **PsychINFO (Ovid)** | exp Postpartum Depression/ | exp Child Psychiatry/ |
| **CINAHL (EBSCO)** | (MH "Depression, Postpartum") OR "maternal depression or postpartum depression or peritnatal depression " OR "antenatal depression" | (MM "Mental Disorders Diagnosed in Childhood+") |
| **Scopus** | TITLE-ABS ("Maternal depression" or "prenatal depression" or "postnatal depression" or "postpartum depression" or "antenatal depression") | TITLE-ABS ("neurodevelopmental disorder" or Autis* or "autism spectrum disorder*" or "autistic disorder*" or "ASD" or "Asperger syndrome" or "pervasive developmental disorder*" or "Rett's syndrome" or "childhood disintegrative disorder*") |
| AB: terms in the abstract; ab:  terms in abstract fields; adj5: terms within five words of each other (any order), ASD: Autism Spectrum Disorder; exp: exploded indexing term; Mesh: Medical Subject Headings; MH: Searches the exact CINAHL Plus Subject Heading, searching both major and minor headings ;MM: Searches the exact CINAHL Subject Heading, searching only in major headings  N5: terms within five words of each other (any order); TI:  terms in the title; TITLE-ABS: terms in the title and abstract, tw: text word search in title  * = truncation  " " = phrase search. | | |

# **eTable 3**. Confounder Variables Accounted by Studies Included in the Systematic Review and Meta-Analysis

| **No** | **Author name** | **Adjusted factors** |
| --- | --- | --- |
|  | Avalos et al. 2023 [1] | Prenatal alcohol use, prenatal tobacco use, pre-pregnancy BMI (kg/m2), birthing parent education, race and ethnicity, age at delivery, marital status, child age at SRS and sex |
|  | Brennan et al. 2023 [2] | Any prenatal antidepressant use, age at visit for the outcome, child’s sex, maternal age at delivery, maternal ethnicity, maternal education status, marital status, prenatal tobacco use, prenatal alcohol use, prenatal marijuana use, and BMI, |
|  | Castro et al. 2016 [3] | Child age at first diagnosis, gender, race, income, mother age at delivery, father age at delivery, mother−government insurance  mother and father educational status, mother past medical history (diabetes mellitus, chronic hypertension), other maternal psychiatric disorders, history of substance/alcohol abuse, and parity |
|  | Chen et al. 2020 [4] | Paternal depression ,family demographic data (income level and residence), parental ages, and sex of children |
|  | Clements et al. 2015 [5] | Child age at first diagnosis, gender, race, birth year, insurance type, maternal age, income tertile, mother age at delivery, father age at delivery, mother - government insurance, mother and father educational status, mother past medical history (diabetes, chronic hypertension), and parity |
|  | Croen et al. 2011[6] | Age, race/ethnicity, education of mother; birth weight, sex, birth year of child, birth facility, and antidepressants use |
|  | Gidaya et al. 2014 [7] | No adjusted factors |
|  | Güneş et al. 2023 [8] | No adjusted factors |
|  | Hagberg et al. 2017 [9] | Child sex, maternal BMi, smoking status, parity, anxiety, antidepressants use, and other psychiatric disorders |
|  | Hviid et al. 2013 [10] | Country of origin, education status, employment status, place of residence, parity, smoking, other maternal psychiatric disorders, and antidepressants use |
|  | Rai et al. 2013 [11] | Parental ages, income, education, occupation, migration status, parity, any other psychiatric condition (including anxiety disorders, affective (bipolar) and non affective psychoses (schizophrenia), somatoform, neurodevelopmental, and personality or drug and alcohol misuse disorders, and paternal depression |
|  | Say et al. 2016 [12] | No adjusted factors |

# **eTable 4.** Quality Assessment of Studies Included in the Final Analysis According to the Newcastle-Ottawa Scale (NOS)

| **R.NO** | **First author, year** | **Quality assessment for cohort** | | | | | | | | | | | | | | | |
| --- | --- | --- | --- | --- | --- | --- | --- | --- | --- | --- | --- | --- | --- | --- | --- | --- | --- |
|  |  | **Selection** | | | | | | | | **Comparator** | | **Outcome** | | | | **Total (9%)** | **Decision** |
|  |  | **Representativeness** | **Selection of non-exposed cohort** | | **Ascertainment of exposure** | | **Demonstration of outcome does not present at the start** | | | **Comparability (**) of cohorts on the design or analysis** | | **Assessment of outcome** | | **Follow-up long enough** | **Adequacy of follow-up** |  |  |
|  | Avalos et al. 2023 [1] | 1 | 1 | | 1 | | 1 | | | 1 | | 1 | | 1 | 1 | 8 | High |
|  | Brennan et al. 2023 [2] | 1 | 1 | | 1 | | 1 | | | 2 | | 1 | | 1 | 1 | 9 | High |
|  | Chen et al. 2020 [4] | 1 | 1 | | 1 | | 1 | | | 1 | | 1 | | 1 | 1 | 8 | High |
|  | Hviid et al. 2013 [10] | 1 | 1 | | 0 | | 1 | | | 2 | | 1 | | 1 | 1 | 8 | High |
| **Quality assessment for case-control studies** | | | | | | | | | | | | | | | | | |
|  | **First author, years** | **Selection** | | | | | | | **Comparator** | | **Outcomes** | | | | | **Total (9%)** | **Decision** |
|  |  | **Adequate case definition (*)** | | **Representativeness of the cases (*)** | | **Selection of Controls (*)** | | **Definition of Controls (*)** | **Comparability of cases and controls (**)** | | **Ascertainment of exposure (*)** | | **Same method of ascertainment (*)** | | **Non-Response rate (*)** |  |  |
|  | Castro et al. 2016 [3] | 1 | | 1 | | 1 | | 1 | 2 | | 1 | | 1 | | 1 | 9 | High |
|  | Clements et al. 2015 [5] | 1 | | 1 | | 1 | | 1 | 1 | | 1 | | 1 | | 1 | 8 | High |
|  | Croen et al. 2011[6] | 1 | | 1 | | 1 | | 1 | 2 | | 1 | | 1 | | 1 | 9 | High |
|  | Gidaya et al. 2014 [7] | 1 | | 1 | | 1 | | 1 | 0 | | 1 | | 1 | | 1 | 7 | Medium |
|  | Güneş et al. 2023[8] | 1 | | 1 | | 1 | | 1 | 0 | | 1 | | 1 | | 1 | 7 | Medium |
|  | Hagberg et al. 2017 [9] | 1 | | 1 | | 0 | | 1 | 2 | | 0 | | 1 | | 1 | 7 | Medium |
|  | Rai et al. 2013 [11] | 1 | | 1 | | 1 | | 1 | 2 | | 1 | | 1 | | 1 | 9 | High |
|  | Say et al. 2016 [12] | 1 | | 1 | | 1 | | 1 | 0 | | 1 | | 1 | | 1 | 7 | Medium |
| Key: For Cohort and case studies: High quality: NOS score above or equal to 8; Medium quality: NOS score of 5 and 7 and Low quality: NOS score below 5  For cross-sectional studies: very good quality (scored 9-10), good quality (scored 7-8), satisfactory quality (scored 5-6), and unsatisfactory studies (0-4 points) | | | | | | | | | | | | | | | | | |

# **References**

1. Avalos LA, Chandran A, Churchill ML, Gao X, Ames JL, Nozadi SS, et al. Prenatal depression and risk of child autism-related traits among participants in the Environmental influences on Child Health Outcomes program. Autism Research. 2023;16(9):1825-35.

2. Brennan PA, Dunlop AL, Croen LA, Avalos LA, Salisbury AL, Hipwell AE, et al. Prenatal Antidepressant Exposures and Autism Spectrum Disorder or Traits: A Retrospective, Multi-Cohort Study. Research on Child and Adolescent Psychopathology. 2023;51(4):513-27. doi: 10.1007/s10802-022-01000-5.

3. Castro VM, Kong SW, Clements CC, Brady R, Kaimal AJ, Doyle AE, et al. Absence of evidence for increase in risk for autism or attention-deficit hyperactivity disorder following antidepressant exposure during pregnancy: a replication study. Transl Psychiatry. 2016;6(1):e708. Epub 20160105. doi: 10.1038/tp.2015.190. PubMed PMID: 26731445; PubMed Central PMCID: PMCPMC5068870.

4. Chen L-C, Chen M-H, Hsu J-W, Huang K-L, Bai Y-M, Chen T-J, et al. Association of parental depression with offspring attention deficit hyperactivity disorder and autism spectrum disorder: A nationwide birth cohort study. Journal of Affective Disorders. 2020:109-14. doi: doi:. PubMed PMID: rayyan-989041910.

5. Clements CC, Castro VM, Blumenthal SR, Rosenfield HR, Murphy SN, Fava M, et al. Prenatal antidepressant exposure is associated with risk for attention-deficit hyperactivity disorder but not autism spectrum disorder in a large health system. Mol Psychiatry. 2015;20(6):727-34. Epub 20140826. doi: 10.1038/mp.2014.90. PubMed PMID: 25155880; PubMed Central PMCID: PMCPMC4427538.

6. Croen LA, Grether JK, Yoshida CK, Odouli R, Hendrick V. Antidepressant use during pregnancy and childhood autism spectrum disorders. Arch Gen Psychiatry. 2011;68(11):1104-12. Epub 20110704. doi: 10.1001/archgenpsychiatry.2011.73. PubMed PMID: 21727247.

7. Gidaya NB, Lee BK, Burstyn I, Yudell M, Mortensen EL, Newschaffer CJ. In utero exposure to selective serotonin reuptake inhibitors and risk for autism spectrum disorder. J Autism Dev Disord. 2014;44(10):2558-67. doi: 10.1007/s10803-014-2128-4. PubMed PMID: 24803368.

8. Güneş H, Tanıdır C, Doktur H, Yılmaz S, Yıldız D, Özbek F, et al. Prenatal, perinatal, postnatal risk factors, and excess screen time in autism spectrum disorder. Pediatrics International. 2023;65(1):1-7. doi: 10.1111/ped.15383. PubMed PMID: 174473618. Language: English. Entry Date: 20240101. Revision Date: 20240101. Publication Type: Journal Article.

9. Hagberg KW, Robijn A, Jick SS. Maternal depression and antidepressant use during pregnancy and risk of autism spectrum disorders in offspring: Population-based cohort and bidirectional case-crossover sibling study. Pharmacoepidemiology and Drug Safety. 2017;26:470. doi: doi:. PubMed PMID: rayyan-989042888.

10. Hviid A, Melbye M, Pasternak B. Use of selective serotonin reuptake inhibitors during pregnancy and risk of autism. N Engl J Med. 2013;369(25):2406-15. doi: 10.1056/NEJMoa1301449. PubMed PMID: 24350950.

11. Rai D, Lee BK, Dalman C, Golding J, Lewis G, Magnusson C. Parental depression, maternal antidepressant use during pregnancy, and risk of autism spectrum disorders: population based case-control study. Bmj. 2013;346:f2059. Epub 20130419. doi: 10.1136/bmj.f2059. PubMed PMID: 23604083; PubMed Central PMCID: PMCPMC3630989.

12. Say GN, Karabekiroğlu K, Babadağı Z, Yüce M. Maternal stress and perinatal features in autism and attention deficit/hyperactivity disorder. Pediatr Int. 2016;58(4):265-9. Epub 20160202. doi: 10.1111/ped.12822. PubMed PMID: 26338105.
